# Comparing mortality risk of patients with acute hip fractures admitted to a major trauma centre on a weekday or weekend

**DOI:** 10.1038/s41598-017-01308-z

**Published:** 2017-04-27

**Authors:** Rajpal Nandra, Jack Pullan, Jonathan Bishop, Khalid Baloch, Liam Grover, Keith Porter

**Affiliations:** 1 Orthopaedic Specialist Trainee, West Midlands Deanery, Birmingham, UK; 2 Medical Student, University of Birmingham, Birmingham, UK; 3 National Institute of Health Research (NIHR) Surgical Reconstruction & Microbiological Research Centre (SRMRC), Birmingham, UK; 4 Consultant Orthopaedic Surgeon, University hospital Birmingham, Birmingham, UK; 5 Professor of Biochemical Engineering, University of Birmingham, Birmingham, UK; 6 Professor of Traumatology, University of Birmingham, Birmingham, UK

## Abstract

Proximal femoral fractures are a major public health concern with estimated annual direct and social costs amounting to £2 billion and average 30-day mortality risk of 7.5%. In response to the recent debate over out-of-hours hospital provision we investigated the ‘weekend effect’ at a major trauma centre, caring for acute injuries. A single centre, multi-surgeon review of 2060 patients performed. The distribution of patient and treatment variables compared in patients admitted on a weekday or the weekend. Fewer patients met performance indicators during weekend admission, time to surgery (63 vs. 71%) and time to geriatric review (86 vs. 91%). Weekend admission 30-day mortality was marginally lower than weekday (9.7% vs. 10.2%, OR 0.94, 95% CI 0.67 to 1.32, p = 0.7383). Increasing age, female gender, co-morbidities and confusion increased mortality risk. Binary regression analysis including these variables found no significant ‘weekend effect’. Despite the unit observing an increasing workload in the last five years, with meticulous workforce planning, senior doctor provisions and careful use of resources, it is possible to provide a seven-day fracture neck of femur service with no variation in thirty-day mortality by the day of admission.

## Introduction

Proximal femoral fractures affect geriatric patients with multiple comorbidities and poor physiological reserve. This renders patients vulnerable and a third will die within the anniversary of their injury^1^. In 2014, the national hip fracture database reported an overall 30-day mortality (TDM) of 7.5% in England, Wales and Northern Ireland for patients with acute proximal femoral fracture (PFF)^1^. It is not uncommon for individual hospitals to report higher TDM in the region of 10 to 12%^2^. In other medical specialities, patients presenting with acute perforated appendix, bacteraemic community acquired pneumonia or ruptured abdominal aortic aneurysm, have TDM rates of 0.5%, 30% and 37% respectively^3–5^. There is also evidence to support inferior outcomes with weekend admission and surgery in these pathologies^6^. Freemantle *et al*. report a ‘weekend effect’ with higher relative risk of 30-day mortality (10 vs 15%)^7^. The findings of this study are widely debated, with newer evidence emerging to tackle criticisms of causality and staffing levels^8, 9^. Meacock *et al*. found no difference with emergency department admissions but did observe lower admission rates at the weekend and when subgrouping by diagnosis, higher weekend mortality was attributed to severity of illness^10^. There is evidence to suggest higher weekend mortality differentiated by diagnosis in emergency attendances and neonatal admissions^11, 12^.

Mortality risk is elevated for two decades following a PFF, predominantly from cardiovascular events or pneumonia^13, 14^. As our population ages PFF have become a public health concern with projected U.K. annual incidence of 101,000 in 2020 and yearly healthcare costs of £2 billion^15^. In recognition of treatment needs and frailty of patients we have seen the implementation of a national hip fracture registry, management guidelines and financial incentives for healthcare trusts that comply with efficiency targets^16, 17^. The policies have imparted an ethos of multi-disciplinary care, early surgery and targeted rehabilitation, with a notable reduction in TDM^14^. However, we do not know whether the day of admission influences patient outcomes and overall survival^18, 19^.

Proximal femoral fracture patients offer a valid and relevant population to evaluate the ‘weekend effect’, using short-term survival as proxy outcome to compare weekday and weekend service provision. In light of recent debate concerning out of hours’ emergency provision we investigate the mortality rates of patients admitted with acute PFF managed at a major trauma centre.

## Methods

All patients with a newly diagnosed PFF were identified from a single institutions prospectively maintained database between 2010 and 2015. The study includes 2061 patients with acute fractures. For purpose of analysis the following data were extracted: patient’s age and sex; date of admission; fracture classification; co-morbidity; mental capacity; treatment modality; time to surgery and geriatric assessment; date of death if applicable.

Day of admission was calculated from the date and time of admission to the emergency department. Weekend was defined from midnight Friday to midnight Sunday. Fractures were classified by appearance on plain radiographs into extra or intracapsular^20^. The American Society of Anaesthesiologists (ASA) physical status classification system, routinely recorded for all patients undergoing operative treatment, was used as a surrogate measure of co-morbidities and anaesthetic risk. A ten-point abbreviated mental test (AMT) assessment was performed at the time of admission to assess cognitive function. Time of emergency department attendance defined the starting point for the continuous variables, time to surgery (hours) and geriatric assessment (hours). Pre-operative mobility was sub-classified into independent or use of one walking aid, use of two walking aids/frame or some indoor mobility but never goes outside without help and wheelchair bound or bed bound with no functional mobility. Patient’s outcome and mortality was verified by the office of national statistics to ensure all patients with TDM are identified irrespective of discharge destination or location at the time of death.

Our unit is a tertiary referral centre serving nearly 800,000 patients every year, for civilian patients with additional military provisions. The trust has a treatment pathway directing prompt haemodynamic resuscitation and analgesia, early clinical assessment and radiological diagnosis and definitive management within 36 hours of attendance. Patients are offered an ilio-fasical block in the emergency department following diagnostic radiographs.

Care is co-ordinated by trauma nurse practitioners who work every day, a trauma foundation one doctor, core surgical doctor, orthopaedic registrar and nominated consultant. Patients are admitted to a dedicated trauma ward for pre-operative optimisation, surgical and geriatric assessment. On weekdays, consultant led morning hip fracture operating lists run in addition to a designated trauma list. At weekends a consultant led two-session trauma list will prioritise hip fracture surgery providing there are no trauma patients requiring time critical intervention due to life or limb threatening injuries.

Ward patients are assessed daily by an orthopaedic registrar who leads a multidisciplinary team, including weekends. Ortho-geriatric services are provided by two consultants who review patients on weekdays. On the weekends attending medical registrars and consultant anaesthetists will review patients. Surgery is performed in line with national guidance preferring cemented modular stemmed hemiarthroplasty for intracapsular fractures, or total hip arthroplasty for patients fitting NICE criteria. We take a pragmatic approach to extracapsular fractures using dynamic hip screw fixation or cephalo-medullary devices in most cases. The ethos of our anaesthetic department is to augment general anaesthesia with a spinal or regional block, thus enhancing recovery and early mobilisation. Patients routinely have peri-operative antibiotics. Discharge destination post-surgery varies from rehabilitation wards within the trust, intermediate care centres to home with a safe package of care.

For the power analysis we identified a 3% increase in mortality risk to be significant using outlier evidence from the hip fracture registry^1, 2^. The parameters used were; dichotomous endpoint; two independent sample groups; alpha 0.05 and power of 80%. To detect a statistical difference we need to recruit 1745 weekday and 524 weekend admissions.

For analysis, we used IBM SPSS Statistics Version 20(Armonk, New York) software with a pre-determined significance level of 0.05. Continuous variables underwent tests for normality. A Shapiro-Wilkes test (p > 0.05), visual inspection of histograms and box plots showed that age was not normally distributed, with a skewness of −0.944 (SE 0.63) and kurtosis of 1.009 (SE 0.125) for patients who survived and a skewness of −0.667 (SE 0.187) and kurtosis of −0.761 (SE 0.371) for patients who died. Time to surgery and time to geriatric review also had skewed distributions.

In the first instance, we investigated the credibility of each candidate feature by performing univariate tests. A Chi-square test was used for categorical data. To compare median distributions we used Mann Witney U-tests with a predetermined significance level (p < 0.05). By accepting the null hypothesis (p > 0.05) we can assume no difference in distribution between the candidate feature and day of admission, Table 1. These variables were nominated to assess the effect on 30-day mortality risk (dependant variable), comparing the prevalence of factors (independent variables) in patients dying within thirty days to those surviving using a binomial logistic regression model. The model will assess the effect of changing weekday to weekend admission on 30-day mortality whilst keeping other candidate features constant and generate odd ratios.

**Table 1:** The frequency of patient, fracture and treatment variables in patients admitted during a weekday and weekend.

Variable	Weekday	%	Weekend	%	Sig.
**Year of admission**					
2011	267	17.2	96	19	
2012	312	20.1	80	15.8	
2013	325	20.9	106	21	
2014	304	19.5	122	24.2	
2015	348	22.4	101	20	0.056
**Gender**					
Male	463	29.8	136	26.9	
Female	1093	70.2	369	73.1	0.224
**Age**					
Median years	84	(IQR 11.2)	84.4	(IQR 13.9)	0.914
**ASA Grade**					
1	45	3.8	8	2	
2	373	31.9	111	27.4	
3	589	50.3	221	54.6	
4	160	13.7	64	15.8	
5	4	0.3	1	0.2	0.127
**Median AMTS**	8.5	(IQR 6)	9	(IQR 5)	0.123
**Time to geriatric review (h)**	26.7	(IQR 44.5)	41	(IQR 28.6)	0.992
**Time to surgery (h)**	23.3	(IQR 21.2)	26.5	(IQR 25.4)	0.082
**Fracture Type**					
Intertrochanteric	674	43.3	229	45.3	
Subtrochanteric	73	4.7	23	4.6	
Intracapsular undisplaced	353	22.7	107	21.2	
intracapsular displaced	450	28.9	144	28.5	0.161
**Surgical Fixation**					
Dynamic Hip Screw	559	35.9	209	41.4	
Internal Screw Fixation	39	2.5	9	1.8	
Intramedullary Nail	285	18.3	77	15.2	
Hemiarthroplasty	759	36	166	32.9	
Total Hip Replacement	68	4.4	27	5.3	
Non-operative	44	2.9	17	3.4	0.001
**30 day mortality**					
Alive	1397	89.8	456	90.3	
Dead	159	10.2	49	9.7	0.738

Abbreviations: ASA = American Society of Anaesthesiologists, IQR = interquartile range, h = hours.

Study approval was gained via the local institutional review board (University hospital Birmingham audit committee). Anonymised patient and treatment information from the trust’s neck of femur database was made available for a service evaluation study. The trusts audit policies were adhered to for this study and no patients were directly contacted.

## Results

The study included 2061 patients with mean age of 82.4 years (S.D. 9.84) and 71% of female sex. On average, 412 patients were treated each year and we observed an average annual increase of 22 cases. The mean 30-day mortality was 10.1%, during 2013 we observed the lowest mortality risk where the hospital treated 426 patients with 46 deaths (7%).

A quarter of patients attended on the weekend (505). Table 1 describes the prevalence of variables and patient characteristics for patients admitted on a weekday or weekend. There was no significant difference in distribution of gender, age, anaesthetic risk and location of fracture relative to hip capsule, thus we can assume the two groups were comparable in these demographics.

**Figure 1:**
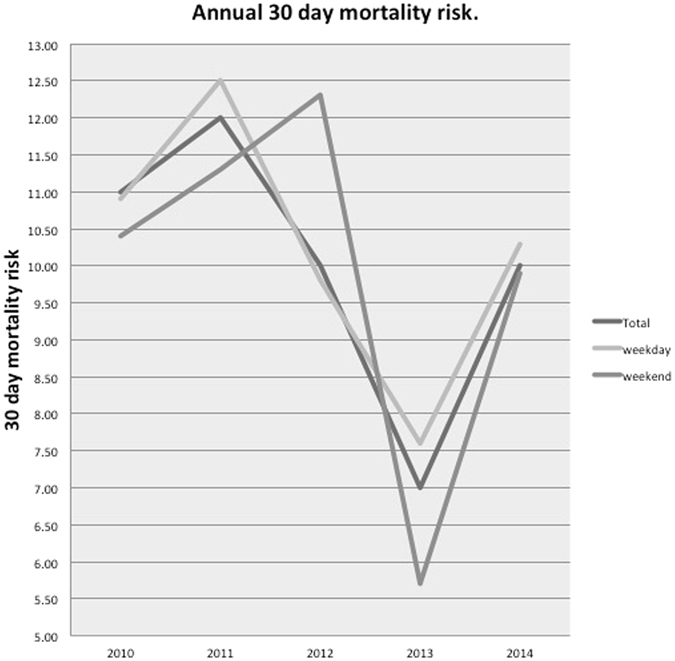
A line chart showing the yearly 30-day mortality risk of all patients, weekday admission and weekend admissions. Weekend mortality is lower than weekday with the exception of 2012.

Over the entire study period weekend mortality was marginally lower than weekday (9.7% vs. 10.2%, OR 0.94, 95% CI 0.67 to 1.32 and P = 0.7383). The mean difference over five years was 0.3% (SD 1.7). Only in 2012 did weekend admission have a higher mortality risk, Fig. 1.

**Figure 2:**
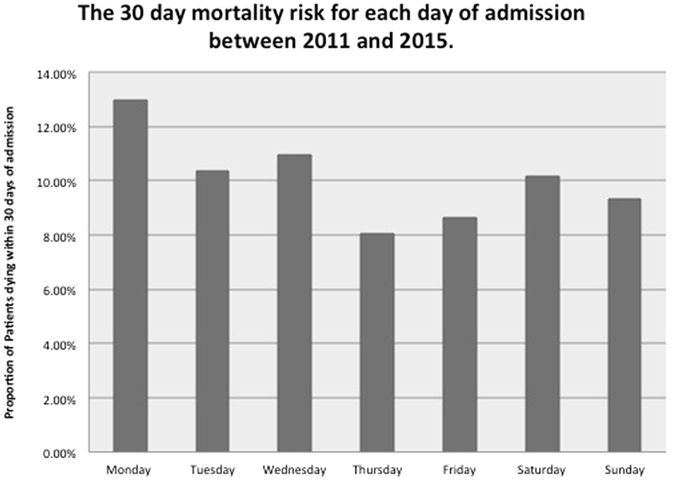
A bar chart illustrating patient mortality risk varied by day of admission between 2010 and 2015.

Over the study period the highest number of patients were admitted on a Monday (324 patients) whilst Saturday had the lowest attendance (226 patients, 10.18%). Figure 2 charts the mortality rates by day of admission, with range of 5% change in risk by day of admission (12.96 vs 8.06%).

The average time to surgery was longer at weekends (38.1 vs 34.8 hours) and only 63% met the 36-hour target for surgery compared to 71% on a weekday. There was no difference in mean time to geriatric assessment (44.6 vs 44.5 hours), however weekend admissions were less likely to adhere to the 72-hour target for review (90.9 vs 86.1%). Across the two groups AMT completion was comparable (76%). Uni-variate analysis of AMT suggested a higher risk of mortality with cognitive decline, AMT <7, OR 2.6 (1.82 to 3.71, p = 0.001).

Patients who mobilised with a wheelchair were twice as likely to die compared to those independently mobile or with aids, OR 2.036 (1.48 to 2.80, P = 0.001).

Multivariate analysis tests the effect of changing one independent variable, whilst keeping the other independent variables static, Table 2. In the final multivariate analysis 970 cases were included as patients with missing data were excluded. In the final data set weekend mortality was 7.5% (23 patients) compared to weekday 7.2% (60 patients), observing 83 deaths in total. Gender and ASA paralleled the original dataset. Age was similar, averaging 83 years and weekend admission had 4 hours delay in time to surgery and a two hour delay for geriatric reviews. The final test set matched the original data set well.

**Table 2:** Binomial logistic regression of 970 acute admissions comparing variables in patients who died within 30 days of admission and survived.

Feature	Variable	Odds Ratio	95% C.I.	p-value
Sex	Male	—	—	
	Female	1.775	(1.061, 2.968)	0.0287
Age	Years	1.057	(1.023, 1.092)	0.0009
ASA	1 and 2	—	—	
	3 and 4	2.231	(1.167, 4.266)	0.0152
Day of admission	Weekday	—	—	
	Weekend	0.939	(0.539, 1.638)	0.8257
AMT	≤7	—	—	
	≥8	2.06	(1.237, 3.429)	0.0055
Pre hospital mobility	Independent ± one aid	—	—	
	Independent: 2 aids or frame	1.103	(0.638, 1.907)	0.7252
	Immobile	0.808	(0.269, 2.424)	0.7031
Time to surgery	Hours	1.007	(1.001, 1.013)	0.0301
Time to geriatric assessment	Hours	0.992	(0.982, 1.001)	0.0783

Abbreviations: ASA = American Society of Anaesthesiologist, AMT = Abbreviated Mental Test.

The output suggests that patients have a significantly greater risk of dying for a one-year increase in age, with a 5.7% increase in odds for every year. We categorised AMT and found that the odds ratio of dying within 30 days of injury is double with cognitive impairment when adjusted for other risk factors. Multivariate analysis suggested that being female (OR 1.76) and having pre-existing co-morbidities (OR 2.23) was associated with higher mortality risk. Increased time to surgery was significantly associated with raised mortality risk, although there was a trend for earlier geriatric assessment to be beneficial this was not significant.

## Discussion

Out of hours emergency cover to admit acute proximal femoral fracture (PFF) patients is necessary twenty-four hours a day. Hospitals, knowing the frailty of these patients, prioritise early investigation and treatment. Our findings suggest that care for acute PFF is similar for patients admitted on a weekday and weekend. The study illustrates the successful implementation of consultant led multi-disciplinary treatment that neutralises any potential ‘weekend effect’. We also identified independent prognostic factors that correlate with short-term survival: increasing age; female gender; co-morbidities and cognitive decline. These can be used to risk assess patients on admission.

The crude mortality estimates over five years were comparable (9.7 vs 10.2). The small reduction in mortality when admitted at the weekend was not statistically significant and clinically this amounts to two fewer deaths a year. Larger studies across multiple sites would be required to provide a generalisable output. Our study lacked power with the final model only recruiting 970 patients. Hence the discriminatory power of the final model was average, with area under the curve (AUROC) of 0.737 (95% CI 0.682–0.792). However, relevant studies have similar accuracy when generating mortality predictors for PFF^2^. Looking at the binary regression output, the model generates two predicted deaths, whilst we observed 83 deaths in the final data set. This suggests there are influential factors not accounted for by the variables in this model and we are missing predictive ability.

There has been an expanse of evidence in 2016 across a range of specialities refuting the weekend effect. To date, evidence for PFF patients has found variability in mortality by day of admission, being higher on the weekend^21–23^. Thomas *et al*.^21^ attributes this to junior anaesthetic cover and no ortho-geriatrician provision on weekends. The study did not control for pre-morbid function or cognitive impairment, which have previously been reported as independent prognostic factors^24^. Research investigating hip fracture outcomes that omit independent variables, such as mobility assessment, are prone to confounding^22^. Being a major trauma centre we staff a dedicated weekday morning hip fracture theatre list supervised by consultants. The department has dedicated trauma nurses to coordinate care and we also prioritise neck of femur patients on weekend trauma lists. This minimises surgical delays and ensures perioperative multi-disciplinary care targets are met. In our study, on weekends, we observed a greater proportion of patients receiving dynamic hip screws for extra-capsular fractures which are considered a lesser surgical insult than cemented hemi-arthroplasty for intra-capsular fractures. We also noted more patients admitted on the weekend had total hip replacements, which are reserved for younger, active individuals according to NICE guidance in 2012.

The factors we found to be associated with poor outcomes in patients with acute PFF are similar to those reported in existing literature: increasing age; female gender; presence of co-morbidities increasing anaesthetic risk and cognitive impairment^2, 25, 26^. Altered biochemistry such as low albumin or low 25-hydroxyvitamin D are also poor prognostic indicators^25^. Impaired pre-operative mobility, diabetes and residing in social care also raises mortality risk^27^. Frost *et al*.^2^ found congestive cardiac and liver disease to moderate survival the most, RR of 3.02 and 4.75 respectively. For every 10 years increase in age, mortality risk doubled (RR 1.92)^2^. The Nottingham hip fracture score encompasses seven variables with the largest magnitude of effect on the outcome of interest, 30-day mortality^28^. Validation studies found a difference of 10.2% in TDM between low and high risk patients^29^. Future studies can use the scoring system to risk profile.

By adjusting for co-morbidities Grimes *et al*.^30^ failed to demonstrate a difference in mortality with delayed surgery (>96 hours)^30^. Moran *et al*.^31^ found no harmful effect when surgery was performed within 4 days. However, any delay secondary to medical co-morbidities elevated mortality risk (17% TDM risk, HR 2.3)^31^. One can conclude from previous studies that outcomes are linked to the overall health of individual patients. The presence of co-morbidities often correlates to age and cognitive function and requires optimisation from senior physicians, with potential to delay surgery. A comprehensive orthogeriatic service reduces mortality and length of stay^32^. Our results suggest that the TDM odds increase for every extra hour taken until the patient has surgery and decrease for every extra hour taken before a geriatric assessment is made. This proposes that patients with increased risk are identified and reviewed sooner by medical physicians and subsequently may have a delay to surgery in order to resuscitate physiological parameters.

We believe the “weekend effect” is complex and speciality dependant. Acute conditions requiring time critical diagnosis or intervention may be hampered by deficient resources and staffing at weekends^18, 33^. Generic studies may not account for the heterogeneity of emergency provisions across specialities and may explain the equipoise in evidence. Care pathways for PFF patients are not time critical^34^. Although a less fragile group, patients with biliary colic have many similarities to PFF patients. Early diagnosis and prompt biliary drainage improves outcomes, but these are not time critical. Other factors were found to have a greater impact on survival (development of cholangitis and dehydration), perhaps why the authors also failed to demonstrate a weekend effect^35^.

Our findings must be considered in light of the studies limitations as a retrospective observational study and the associated discrepancies with data collection, missing data and irregularities in documentation, despite meticulous effort to analyse reliable data fields with consistent definitions. Due to missing data fields the final statistical analysis comprised 970 cases, less than half of the original study sample and was underpowered. This has influenced the predictive ability and accuracy of our analysis. No attempt was made to synthesise missing data.

As described in the methodology, the studied population was treated at a major trauma centre with seven day working rotas, involving consultant led care in orthopaedics, anaesthesia and geriatrics. This may not be achievable in lesser hospital tiers and limits the generalisability of our conclusions. We also recognise the limitations of grading systems, such as ASA. The classification is known to have inter-observer variability. We used it as a proxy for co-morbidities as these were not reliably recorded on the hip fracture database. A prospective study would facilitate the capture of data such as co-morbidities, cardiovascular health and liver disease, reducing bias. We selected thirty-day mortality as it is reliably recorded as we do not routinely follow-up PFF patients, however it is an arbitrary time point and we cannot comment on the weekend effect on longer-term survival.

## Conclusion

With an aging population and increasing prevalence of proximal femoral fractures, it is important that we allocate appropriate resource and staffing to accommodate needs. The unit investigated provides seven-day consultant led multi-disciplinary care for complex injuries involving frail and vulnerable elderly patients. The study failed to demonstrate a weekend effect. A larger multi-centre study is required to generate a generalisable outcome. We successfully identified independent prognostic factors that increase short-term mortality; Increasing age, female gender, higher ASA grade and cognitive decline. With meticulous service provision and robust care pathways we can provide effective seven-day care.
